# Chronic Trazodone and Citalopram Treatments Increase Trophic Factor and Circadian Rhythm Gene Expression in Rat Brain Regions Relevant for Antidepressant Efficacy

**DOI:** 10.3390/ijms232214041

**Published:** 2022-11-14

**Authors:** Lucia Carboni, Laura Rullo, Francesca Felicia Caputi, Serena Stamatakos, Sanzio Candeletti, Patrizia Romualdi

**Affiliations:** Department of Pharmacy and Biotechnology, Alma Mater Studiorum University of Bologna, 40126 Bologna, Italy

**Keywords:** antidepressant, trazodone, BDNF, TrkB, FGF-2, FGFR2, CREB, *Per1*, *Per2*, *Bmal1*

## Abstract

Trazodone is an efficacious atypical antidepressant acting both as an SSRI and a 5HT2A and 5HT2C antagonist. Antagonism to H1-histaminergic and alpha1-adrenergic receptors is responsible for a sleep-promoting action. We studied long-term gene expression modulations induced by chronic trazodone to investigate the molecular underpinning of trazodone efficacy. Rats received acute or chronic treatment with trazodone or citalopram. mRNA expression of growth factor and circadian rhythm genes was evaluated by qPCR in the prefrontal cortex (PFCx), hippocampus, Nucleus Accumbens (NAc), amygdala, and hypothalamus. CREB levels and phosphorylation state were evaluated using Western blotting. BDNF levels were significantly increased in PFCx and hippocampus by trazodone and in the NAc and hypothalamus by citalopram. Likewise, TrkB receptor levels augmented in the PFCx after trazodone and in the amygdala after citalopram. FGF-2 and FGFR2 levels were higher after trazodone in the PFCx. The CREB phosphorylation state was increased by chronic trazodone in the PFCx, hippocampus, and hypothalamus. *Bmal1* and *Per1* were increased by both antidepressants after acute and chronic treatments, while *Per2* levels were specifically augmented by chronic trazodone in the PFCx and NAc, and by citalopram in the PFCx, amygdala, and NAc. These findings show that trazodone affects the expression of neurotrophic factors involved in antidepressant responses and alters circadian rhythm genes implicated in the pathophysiology of depression, thus shedding light on trazodone’s molecular mechanism of action.

## 1. Introduction

Trazodone is an efficacious antidepressant available for the treatment of major depressive disorder (MDD) with or without anxiety since the 1970s [1,2,3]. The efficacy is based on more than one mechanism of action, and the pharmacological profile has not been completely understood until now. Trazodone can inhibit serotonin transporters similarly to serotonin-selective re-uptake inhibitors (SSRIs). In addition, it belongs to the first dual acting antidepressants by also binding to 5HT2A and 5HT2C receptors with an antagonist profile [1,2,4]. Trazodone’s binding affinity for 5HT2A receptors is much higher in comparison with binding affinity for serotonin transporters, therefore at low doses the latter may not be adequately targeted [1]. Blocking of 5HT2B and 5HT1D also contribute to trazodone efficacy [3]. Trazodone actions on 5HT1A receptors and serotonin transporters are suggested to generate a synergistic effect, which potentiates antidepressant activity [4,5,6], possibly triggering a faster onset [7]. At antidepressant doses, no effects on cholinergic signalling are reported [1,3]. Moreover, trazodone is converted to an active metabolite endowed with agonistic properties for several serotonin receptors [1]. Furthermore, trazodone acts as an antagonist to H1 histaminergic receptors and alpha 1 adrenergic receptors. This profile is considered responsible for trazodone efficacy as a sleep-inducing agent. At low doses (100 mg) the sedative effect prevails, whereas higher doses (300 mg) are required for antidepressant efficacy [4,8].

In major depressive disorder, trazodone is effective in controlling most symptoms associated with the disease, such as anhedonia, feelings of sadness and hopelessness, low energy levels, and suicidal thoughts [2,3,6,9,10,11]. Tolerability is comparable to other second generation antidepressants [2,5,6,10]. However, characteristically beneficial effects have been demonstrated for trazodone by regularizing sleep parameters [3,12]. In addition, it is reported to be used as adjunct therapy to counteract insomnia, anxiety, and sexual dysfunction associated with SSRI administration [2,5]. Efficacy in insomnia has caused its widespread off-label use as hypnotic agent, especially in the presence of psychiatric or neurological disorders [2,3,5,13]. The effectiveness in improving sleep parameters in the presence of sleep disorders has been shown in Alzheimer’s disease patients [14], in association with delayed cognitive decline [15]. However, increased risk of suicide attempts has been reported for this indication [16].

Besides its therapeutic indication as antidepressant, trazodone is also effective in anxiety disorders such as generalised anxiety disorder, panic disorder, and post-traumatic stress disorder; in obsessive-compulsive disorder; in eating disorders; in substance use disorders; in pain conditions; and in sexual disorders [2,5,9,17].

The clinical response to SSRI and tricyclic antidepressants is reported to require a therapeutic latency period lasting 3–4 weeks before therapeutic benefits are perceived. Likewise, although trazodone inhibitory and antagonistic effects on transporters and neurotransmitter receptors happen within minutes of administration, antidepressant efficacy requires longer timelines. Therefore, it is assumed that long-term molecular adaptations in cellular pathways downstream to receptor binding are necessary to promote clinical responses. Differently from other antidepressants [18,19,20,21,22], very few studies investigated molecular changes caused by trazodone in brain regions involved in depressive symptoms.

Over the past 20 years, a wealth of research demonstrated a link between decreased neurotrophic factors in brain regions involved in limbic regions regulating mood and cognition in association with stress exposure and depression, while antidepressants could reverse these effects [23]. A most prominent role has been demonstrated for BDNF and its receptor TrkB [23]. Compelling evidence demonstrated a crucial role for BDNF signalling in different central nervous system disorders [24,25,26], including major depression, with reduced brain levels in depressive patients’ brains and peripheral tissues [20,27]. Moreover, increased BDNF expression is required to mediate efficacy of all available antidepressant therapies, both as medicines and as electroconvulsive treatment [20,27,28]. Within the signalling pathways involved in eliciting the antidepressant-mediated neuroplasticity responses required for therapeutic efficacy, a prominent role is exerted by the transcription factor CREB [21]. CREB-dependent transcription is activated when the transcription factor is phosphorylated on Ser^133^ by several protein kinases, and this phosphorylation is required for activation. Altered CREB phosphorylation after antidepressant treatment has been demonstrated in a region- and drug-dependent manner [21]. In parallel, more recent discoveries revealed a contribution to neuroplastic responses relevant for antidepressant effects by growth factors belonging to the FGF family [29,30,31,32]. Among those, decreased FGF-2 and FGFR2 expression were found in post-mortem brain of individuals with MDD [31]. In addition, antidepressant treatments can increase FGF-2 levels in cortical neurons in rats and in cultured cortical astrocytes [33,34].

Disruption of the circadian rhythm and derived sleep disturbances are reported to exert a significant influence to precipitate the onset of major depression disease episodes [35,36,37]. Indeed, the ability to rapidly modulate and reset the circadian clock has been associated to the rapid antidepressant response resulting from sleep deprivation or ketamine administration [38,39]. *Bmal1*, *Per1*, and *Per2* belong to the transcriptional–translational feedback loop driving the 24 h rhythmicity of the molecular clock in cells [35]. Altered expression has been reported in peripheral blood cells of MDD patients following escitalopram treatment [40].

The objective of this investigation was to study long-term gene expression modulations induced by chronic trazodone treatment. The findings aim to increase the comprehension of the specific profile of this atypical and valuable antidepressant by revealing molecular mechanisms involved in its antidepressant efficacy. Molecular changes induced by trazodone have been compared with those exerted by citalopram, a typical SSRI agent, to collect data on the neurobiological effects specifically belonging to trazodone in addition to the common ability to bind and inhibit the serotonin transporter.

## 2. Results

### 2.1. Trophic Factors

**Figure 1:**
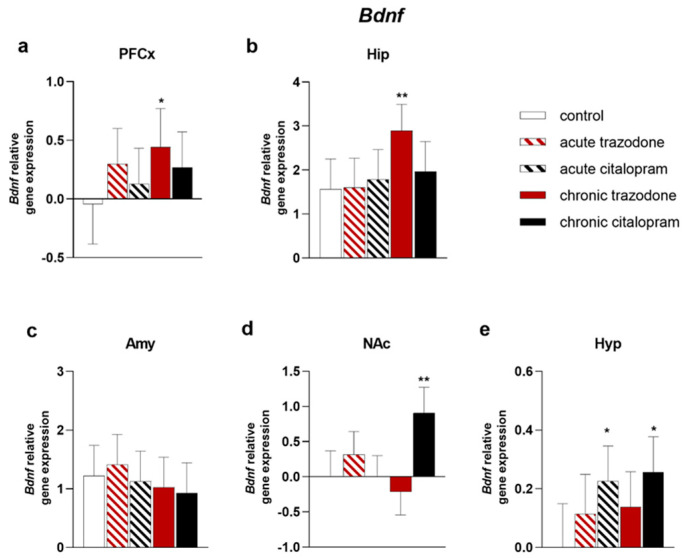
mRNA levels of *Bdnf* in the PFCx (**a**), Hip (**b**), Amy (**c**), NAc (**d**), and Hyp (**e**) after acute or chronic treatment with trazodone, citalopram, or vehicle. The least square predicted means with 95% confidence intervals are plotted. Data were log-transformed when required to stabilise the variance and satisfy the parametric assumptions (**a**,**d**,**e**). **: *p* < 0.01; *: *p* < 0.05 in the Planned Comparison vs. control. *n* = 6/group.

**Figure 2:**
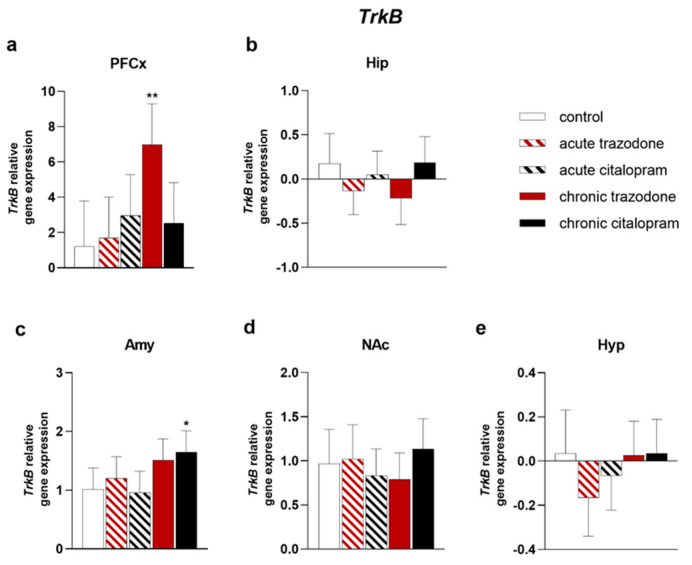
mRNA levels of *TrkB* in the PFCx (**a**), Hip (**b**), Amy (**c**), Nac (**d**), and Hyp (**e**) after acute or chronic treatment with trazodone, citalopram, or vehicle. The least square predicted means with 95% confidence intervals are plotted. Data were log-transformed when required to stabilise the variance and satisfy the parametric assumptions (**b**,**e**). **: *p* < 0.01; *: *p* < 0.05 in the Planned Comparison vs. control. *n* = 6/group.

The expression of trophic factor genes involved in antidepressant efficacy and of their respective receptors was evaluated after treatment with chronic or acute trazodone or citalopram in comparison with control levels in brain regions relevant to MDD. *Bdnf* levels were significantly increased in the pre-frontal cortex (PFCx) (*p* = 0.042, Figure 1a) and in the hippocampus (Hip) (*p* = 0.0068, Figure 1b) after chronic trazodone. Chronic citalopram increased *Bdnf* expression in the Nucleus Accumbens (NAc) (*p* = 0.0018, Figure 1d) and in the hypothalamus (Hyp) (*p* = 0.012, Figure 1e). In the Hyp, *Bdnf* expression was similarly augmented by acute citalopram as well (*p* = 0.023, Figure 1e). Levels of the BDNF receptor *TrkB* were significantly higher in the PFCx of rats treated with chronic trazodone (*p* = 0.0025, Figure 2a); a trend towards decrease was observed in the Hip (*p* = 0.086, Figure 2b) and towards increase in the amygdala (Amy) (*p* = 0.058, Figure 2c). Chronic citalopram increased *TrkB* expression in the Amy (*p* = 0.019, Figure 2c).

**Figure 3:**
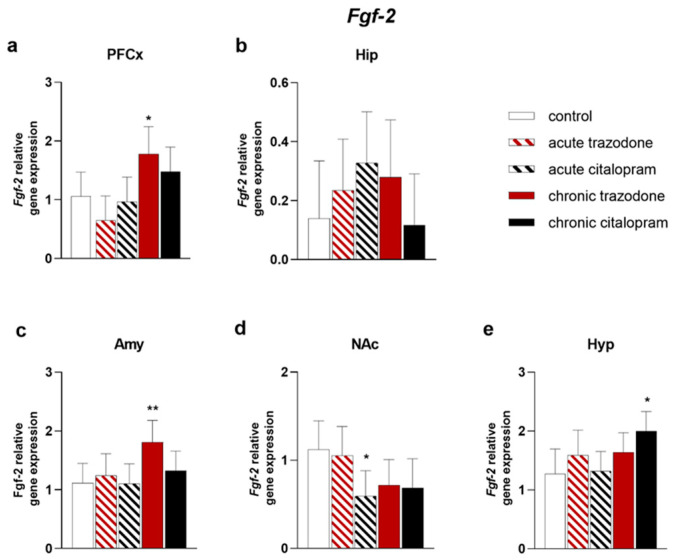
mRNA levels of *Fgf-2* in the PFCx (**a**), Hip (**b**), Amy (**c**), NAc (**d**), and Hyp (**e**) after acute or chronic treatment with trazodone, citalopram, or vehicle. The least square predicted means with 95% confidence intervals are plotted. **: *p* < 0.01; *: *p* < 0.05 in the Planned Comparison vs. control. *n* = 6/group.

**Figure 4:**
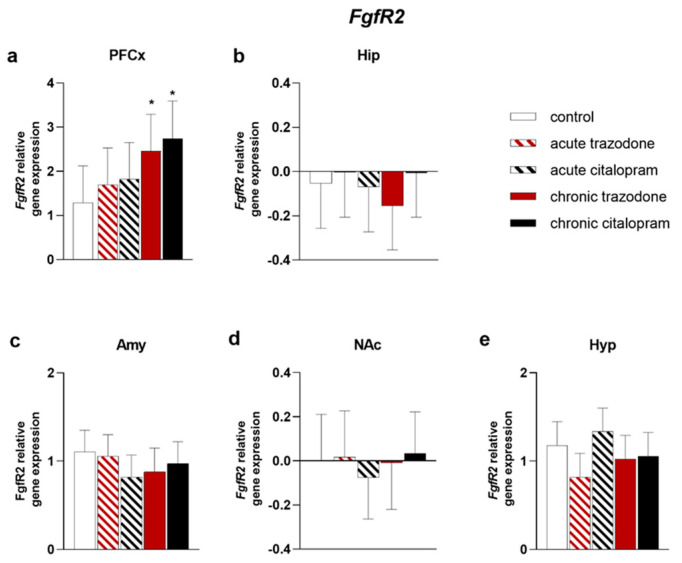
mRNA levels of *Fgfr2* in the PFCx (**a**), Hip (**b**), Amy (**c**), NAc (**d**), and Hyp (**e**) after acute or chronic treatment with trazodone, citalopram, or vehicle. The least square predicted means with 95% confidence intervals are plotted. Data were log-transformed when required to stabilise the variance and satisfy the parametric assumptions (**b**,**d**). *: *p* < 0.05 in the Planned Comparison vs. control. *n* = 6/group.

After chronic trazodone, *Fgf-2* levels were higher in the PFCx (*p* = 0.025, Figure 3a) and Amy (*p* = 0.0093, Figure 3c); a trend for decrease was detected in the NAc (*p* = 0.070, Figure 3d). Chronic citalopram augmented *Fgf-2* expression in the Hyp (*p* = 0.011, Figure 3e). In contrast, in the NAc, acute citalopram significantly reduced *Fgf-2* levels (*p* = 0.021, Figure 3d), while chronic treatment caused a trend for decrease similar to that observed after trazodone (*p* = 0.057, Figure 3d). Administration of both chronic trazodone and citalopram increased *Fgfr2* levels in the PFCx (*p* = 0.037 for trazodone and *p* = 0.017 for citalopram, Figure 4a). A trend for reduced levels was detected in the Hyp after acute trazodone administration (*p* = 0.064). No significant changes were observed in other brain areas (Figure 4).

**Figure 5:**
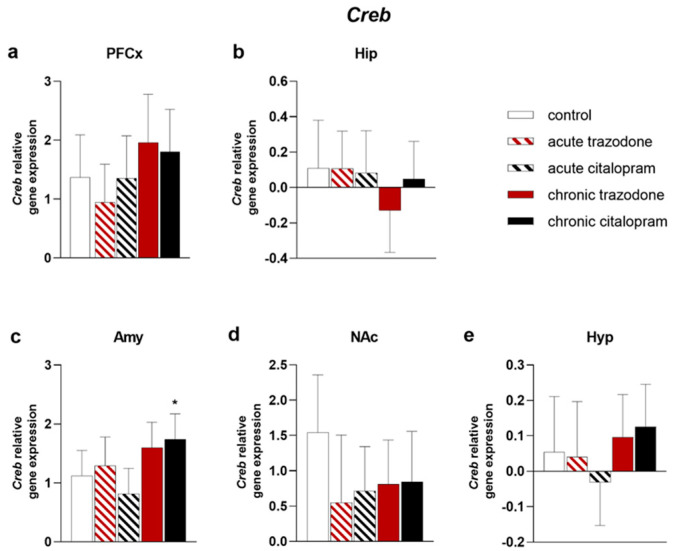
mRNA levels of *Creb* in the PFCx (**a**), Hip (**b**), Amy (**c**), NAc (**d**), and Hyp (**e**) after acute or chronic treatment with trazodone, citalopram, or vehicle. The least square predicted means with 95% confidence intervals are plotted. Data were log-transformed when required to stabilise the variance and satisfy the parametric assumptions (**b**,**e**). *: *p* < 0.05 in the Planned Comparison vs. control. *n* = 6/group.

**Figure 6:**
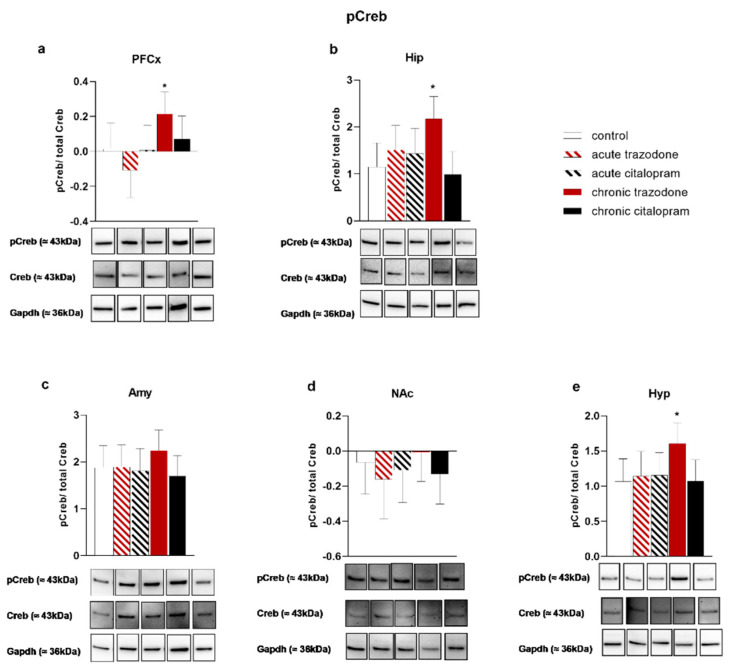
Protein levels of phospho-CREB/total CREB in the PFCx (**a**), Hip (**b**), Amy (**c**), NAc (**d**), and Hyp (**e**) after acute or chronic treatment with trazodone, citalopram, or vehicle. The least square predicted means with 95% confidence intervals are plotted. Data were log-transformed when required to stabilise the variance and satisfy the parametric assumptions (**a**,**d**). Representative blots are shown in each panel; blot images are displayed in Supplementary Figure S2. *: *p* < 0.05 in the Planned Comparison vs. control. *n* = 6/group.

*Creb* expression was augmented by chronic citalopram treatment in the Amy (*p* = 0.048, Figure 5c), while no significant alterations were revealed in other brain regions (Figure 5). No significant changes were observed after trazodone administration (Figure 5). In contrast, the CREB phosphorylation state was significantly increased by chronic trazodone in the PFCx (*p* = 0.048, Figure 6a), Hip (*p* = 0.0059, Figure 6b), and Hyp (*p* = 0.017). CREB protein levels were not altered in any tested condition (Supplementary Figure S1).

### 2.2. Circadian Rhythm Genes

**Figure 7:**
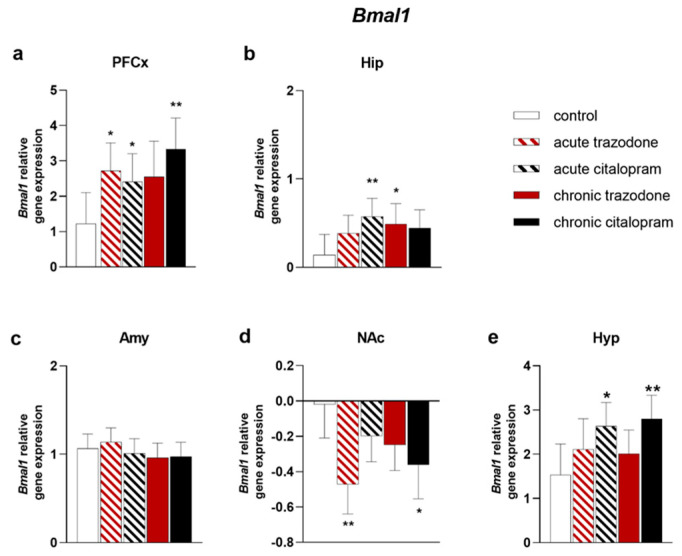
mRNA levels of *Bmal1* in the PFCx (**a**), Hip (**b**), Amy (**c**), NAc (**d**), and Hyp (**e**) after acute or chronic treatment with trazodone, citalopram, or vehicle. The least square predicted means with 95% confidence intervals are plotted. Data were log-transformed when required to stabilise the variance and satisfy the parametric assumptions (**b**,**d**). **: *p* < 0.01; *: *p* < 0.05 in the Planned Comparison vs. control. *n* = 6/group.

Next, we analysed the impact of antidepressant treatments on the expression of circadian rhythm genes. In PFCx, both acute and chronic citalopram treatment augmented *Bmal1* mRNA levels (*p* = 0.048, *p* = 0.0026, respectively; Figure 7a). Likewise, acute and chronic trazodone administrations increased *Bmal1* levels (*p* = 0.016; *p* = 0.054, Figure 7a). Similarly, in the Hip, citalopram both acute and chronic treatments raised *Bmal1* mRNA (*p* = 0.0086; *p* = 0.054, Figure 7b). Trazodone reproduced a similar pattern, with chronic treatment causing a significant increase (*p* = 0.038, Figure 7b). In contrast, *Bmal1* reduction was observed in the NAc after acute or chronic trazodone (*p* = 0.0013; *p* = 0.062, Figure 7d) or after chronic citalopram (*p* = 0.012, Figure 7d). In the Hyp a specific response to citalopram was detected, with both acute and chronic treatments triggering increased *Bmal1* levels (*p* = 0.017; *p* = 0.0076, Figure 7e).

**Figure 8:**
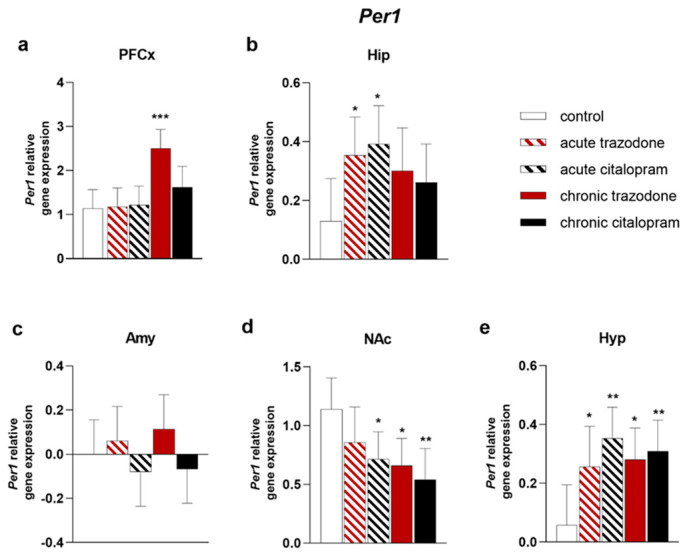
mRNA levels of *Per1* in the PFCx (**a**), Hip (**b**), Amy (**c**), NAc (**d**), and Hyp (**e**) after acute or chronic treatment with trazodone, citalopram, or vehicle. The least square predicted means with 95% confidence intervals are plotted. Data were log-transformed when required to stabilise the variance and satisfy the parametric assumptions (**c**). ***: *p* < 0.001; **: *p* < 0.01; *: *p* < 0.05 in the Planned Comparison vs. control. *n* = 6/group.

Chronic trazodone treatment increased *Per1* in the PFCx (*p* = 0.0001, Figure 8a). In the Hip, significant alterations were only detected after acute treatments; both antidepressants augmented *Per1* levels (trazodone *p* = 0.026; citalopram *p* = 0.011, Figure 8b). Resembling *Bmal1*, in the NAc reduced *Per1* levels were induced by antidepressant treatments (acute and chronic citalopram: *p* = 0.021; *p* = 0.0027, Figure 8d; chronic trazodone: *p* = 0.010, Figure 8d). In the Hyp, a generalised increase was brought about by antidepressant administration, with all treatments causing higher *Per1* levels (acute trazodone *p* = 0.036; chronic trazodone *p* = 0.015; acute citalopram *p* = 0.0024; chronic citalopram *p* = 0.0075, Figure 8e).

**Figure 9:**
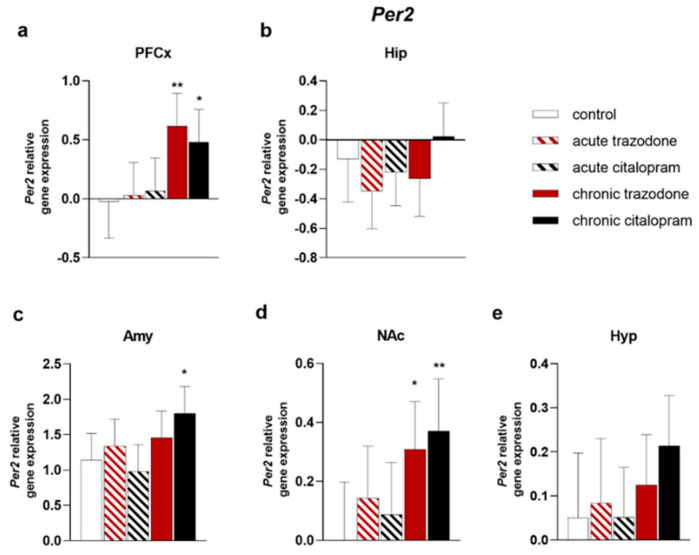
mRNA levels of *Per2* in the PFCx (**a**), Hip (**b**), Amy (**c**), NAc (**d**), and Hyp (**e**) after acute or chronic treatment with trazodone, citalopram, or vehicle. The least square predicted means with 95% confidence intervals are plotted. Data were log-transformed when required to stabilise the variance and satisfy the parametric assumptions (**a**,**b**,**d**,**e**). **: *p* < 0.01; *: *p* < 0.05 in the Planned Comparison vs. control. *n* = 6/group.

In contrast, *Per2* appeared to specifically respond to chronic treatments. In the PFCx, both chronic trazodone and citalopram induced *Per2* increases (*p* = 0.0046; *p* = 0.021, Figure 9a), whereas in the Amy only chronic citalopram augmented *Per2* (*p* = 0.018, Figure 9c). Like the PFCx, in the NAc, chronic trazodone and citalopram triggered higher *Per2* levels (*p* = 0.020; *p* = 0.0086, Figure 9d).

### 2.3. Multivariate Analysis

**Figure 10:**
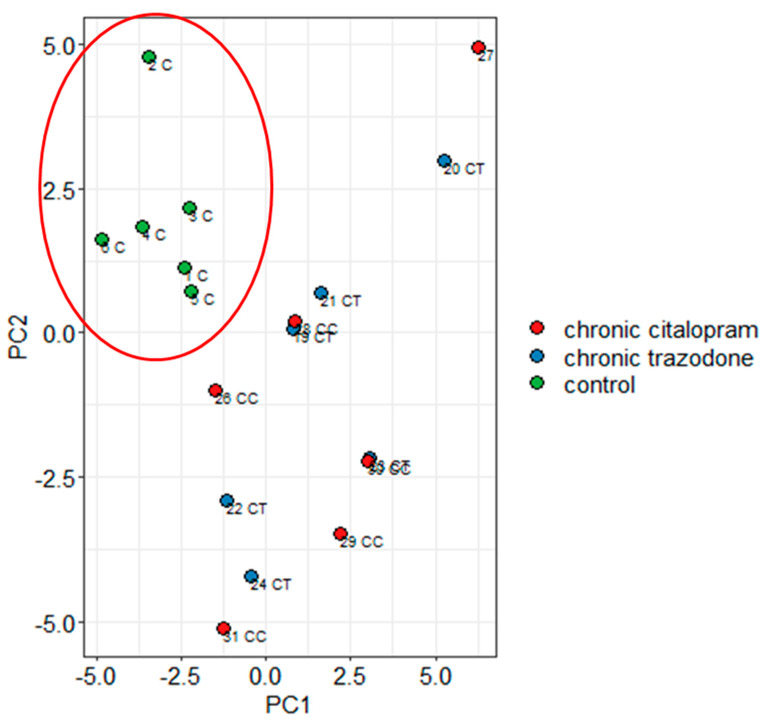
PCA of mRNA or protein levels of all genes/proteins investigated after chronic trazodone, chronic citalopram, or chronic vehicle (control) treatment. A clear separation by antidepressant versus vehicle treatment (circled) is observed by adopting an unsupervised approach.

As a further step, we carried out a multivariate analysis including all gene and protein levels measured in all brain regions after chronic antidepressant treatments and controls. Principal Components Analysis showed that a strong separation based on pharmacological treatment (chronic trazodone or chronic citalopram) was achieved by Principal Component 1 and Principal Component 2 (Figure 10). Variables providing stronger contributions to the Principal Component 1 were hypothalamic levels of *Bmal1*, *Fgf-2*, *Per1*, *Per2*, *Creb*, and *TrkB*, in addition to NAc levels of *Per1*, *Bmal1*, and *Fgf-2* (Supplementary Table S1).

## 3. Discussion

This study aimed at contributing to the comprehension of the neurobiological basis of trazodone efficacy by studying molecular changes engendered by chronic treatment in brain regions relevant for MDD pathophysiology. Two neurotrophic factors involved in the regulation of neural plasticity responses relevant for antidepressant efficacy were our first object of investigation. Subsequently, the impact on the main regulators of circadian rhythms was examined. Overall, our findings show that trazodone increased neurotrophic factor responses, somehow resembling the effects induced by the classic SSRI citalopram, albeit with a number of gene and brain region specificities, mainly in the direction of a more widespread impact. Moreover, we discovered that both antidepressants could influence circadian genes after both acute and chronic administration. These results suggest that circadian gene modulation may exert a role in resetting circadian rhythms which are often dysregulated in MDD. More specifically, *Per2* increase was altered only after chronic antidepressant treatment, implying that this response is possibly associated to therapeutic response. Furthermore, findings from multivariate analysis indicate that antidepressant influence on circadian gene transcription exerted a greater impact than gene expression changes of neurotrophic genes, thus underscoring the relevance of this mechanism for the neurobiological underpinning of the antidepressant response. As a caveat, to strengthen the case for the potential significance of these mechanisms in antidepressant efficacy, further research will be needed in rodent models of disease, since naïve rats were investigated in the present study.

Regarding neurotrophic factors, we found that chronic antidepressant treatments increased *Bdnf* levels. Compelling evidence shows that this growth factor is implicated in the response to antidepressants. Indeed, antidepressant behaviours are attenuated in transgenic mice bearing *Bdnf* deletion, whereas antidepressant-like responses are induced by BDNF injection [27,28]. BDNF function in the antidepressant response is linked to its ability to act as a regulator of synaptic plasticity, neuronal function, and survival in the adult brain [19]. Since chronic stress, a known precipitating factor for MDD, is responsible of causing neuronal atrophy in the hippocampal and prefrontal regions, trophic factor activity is deemed essential to counteract these impairments [19,41]. In this framework, the ability to elicit BDNF increase in forebrain regions can be associated with clinical efficacy. Interestingly, fast acting antidepressants are reported to be able to quickly increase BDNF levels, whereas chronic treatments are required for antidepressants generating delayed therapeutic responses [19]. In this study, we observed increased *Bdnf* expression in the PFCx and Hip after chronic (but not acute) trazodone, whereas citalopram augmented *Bdnf* in the NAc and Hyp. These results are in line with previous findings for what concerns citalopram, where generally no effects on forebrain BDNF were detected when treatments were carried out in naïve animals [42,43,44,45,46]. Contrasting reports described BDNF increase versus no change in the Hip after a definitely higher dose (10 mg/kg escitalopram, which is citalopram’s therapeutically active enantiomer) [47,48]. Citalopram’s influence typically unfolds as the ability to reverse stress-induced BDNF decreases in rodent models of depression based on stress exposure [43,46,49,50,51]. Similar patterns were observed for the BDNF protein [44,52,53,54]. Regarding trazodone, no previous data are available, except for a reported increase in BDNF levels induced by trazodone in human neural stem cells [55]. Although suggesting a similar ability to elicit neurotrophic responses, these results were derived in too different a model to allow a meaningful comparison.

BDNF receptor *TrkB* expression was also increased by chronic trazodone in the PFCx, whereas chronic citalopram augmented it in the Amy. TrkB has been implicated in antidepressant responses in pre-clinical models, both in consequence of BDNF-mediated activation, and as a result of direct binding and activation by the antidepressants themselves [56,57,58]. The most relevant brain areas implicated in this putative efficacy-related response are forebrain regions [28], thus suggesting a potential impact of trazodone-induced *TrkB* rise. Citalopram-stimulated increase in the Amy and a lack of effect in the Hip agree with previous data [48,59], whereas no prior information has been available until now about trazodone.

In addition to neurotrophins, dysregulations in Fibroblast Growth Factors pathways have more recently been involved in the neurobiological underpinnings of MDD [30]. FGFs have been firstly implied in neural development, but a crucial role in neurogenesis and in neuroplasticity in adulthood was subsequently discovered, suggesting neuromodulatory functions in anxiety-like and depression-like behaviours [29,30,31]. Indeed, recent studies in humans revealed altered levels of FGF family members in MDD patients, both in blood and in brain [32]. Moreover, blood FGF-2 protein levels contribute to a peripheral biomarker panel of escitalopram efficacy in MDD patients [60]. However, although available data point to the importance of this family of growth factors in MDD, the results are inconsistent and a clear comprehension of its role in MDD pathophysiology is still missing. Nevertheless, there is evidence that FGF-2 levels are decreased in MDD and increased by antidepressants [30,34,61,62] and that FGF-2 elicits antidepressant-like behaviours [63,64]. In line with these findings, we observed that both trazodone and citalopram were able to increase the expression of *Fgf-2* and *Fgfr2* in the PFCx, Amy, or Hyp.

To investigate a potential mechanism of the increased transcription of trophic factors, we analysed CREB protein phosphorylation on Ser^133^, which is required for CREB activity as a transcription factor. CREB-regulated gene expression has been demonstrated to be crucial for neuroplasticity and survival responses critical for antidepressant efficacy [21,65,66]. The detected increased CREB phosphorylation following chronic trazodone is consistent with proposing this mechanism as responsible of trophic factor responses.

Next, we investigated antidepressant influence on circadian genes. We discovered that both trazodone and citalopram augmented circadian gene expression. Although the increase emerged after both acute and chronic treatments for *Bmal1* and *Per1*, only chronic administration affected *Per2* expression in the PFCx, NAc, and Amy. A paucity of data exists on antidepressant effects on circadian genes. In depressed patients, escitalopram was able to restore *Per2* and *Bmal1* mRNA dysregulations in peripheral blood mononuclear cells, whereas *Per1* was not affected [40]. In a similar clinical study, ramelteon, a melatonergic agonist antidepressant, could resynchronise altered *Per1* and *Per2* mRNA levels [67]. In rodent models of depression, dysregulation of circadian gene expression has been observed, with *Bmal1* and *Per2* as the more affected [68,69,70,71]; normal rhythms could be reinstated after imipramine or melatonin treatment [69,71]. However, no effect could be devised after imipramine treatments in naïve animals [69]. No data are available for other antidepressants, except for modulation by quetiapine, which is used as add-on therapy in MDD, although belonging to the class of antipsychotic agents [72]. Regarding trazodone, to our knowledge this is the first study reporting modifications of circadian genes. In line with our findings, clinical data in Alzheimer’s disease patients showed significant improvement in relative rhythm amplitude after two weeks of trazodone use [73].

In conclusion, trazodone is an efficacious antidepressant with a long clinical history and a specific therapeutic profile. Since its discovery, few data were generated about its long-term effect on brain transcriptional frame. These findings contribute to shed light on trazodone molecular mechanism of action, suggesting that an impact on growth factors and on circadian rhythm genes may contribute to its efficacy.

## 4. Materials and Methods

### 4.1. Animals

Male (*n* = 32) Sprague–Dawley rats (150 g body weight) were purchased from Envigo, Italy. Rats were housed 3–4/cage at a constant room temperature of 21 ± 1 °C and maintained on a 12:12 h light/dark cycle with lights on at 7.30 a.m. with freely available food and water. The experiment started one week after arrival to let the animals acclimatise. The Italian Health Ministry Ethical Committee for Protection of Animals approved the study (approval number: 6/2018-PR) and all procedures were conducted in accordance with the European Communities Council Directive 2010/63/EU and the national D.Lgs 4 March 2014, no. 26. Efforts were performed to reduce the number of animals and minimise their suffering. This study is compliant with ARRIVE guidelines [74].

### 4.2. Treatments

The experimental design encompassed five groups (*n* = 6–7 rats/group) treated by intraperitoneal route. Rats receiving chronic treatments were administered either 28 mg/kg trazodone (Merck Life Science, Milano, Italy) or 6 mg/kg citalopram (Elopram, Lundbeck Italia, Padova, Italy) for 21 days in 2 mL/kg saline solution (Supplementary Figure S3). Doses were selected to correspond [75] to upper limits of human antidepressant therapy ranges (300 mg daily for trazodone and 40 mg daily for citalopram). Acute treatments were carried out by administering saline for 20 days followed by a single administration of either 28 mg/kg trazodone or 6 mg/kg citalopram on day 21st. Controls were treated with saline for 21 days. Rats were sacrificed by guillotine and brain regions were dissected, immediately frozen in dry ice, and stored at −80 °C.

### 4.3. Gene Expression

The real-time quantitative PCR experiments were carried out following previously published methods [76], with some modifications. RNA extraction was performed with the Aurum total RNA fatty and fibrous tissue kit (Bio-Rad, Hercules, CA, USA), which includes a DNase I digestion step, following manufacturer’s instructions. RNA levels were quantified by UV absorbance in a NanoDrop 2000c spectrophotometer (Thermo Fisher Scientific, Waltham, MA, USA). cDNA was synthesised from 1 µg RNA using the iScript Advanced cDNA synthesis Kit (Bio-Rad, Hercules, CA, USA). Real-time PCR was performed by Sybr Green technology in a 7900HT Fast Real-Time PCR System (Applied Biosystems, Thermo Fisher Scientific, Waltham, MA, USA) with SSO Advanced Universal SYBR Green Supermix (Bio-Rad, Hercules, CA, USA) in 20 µL according to this protocol: stage 1: 95 °C, 20 s; stage 2: 40 × (95 °C, 3 s; 60 °C, 30 s). Primers were selected using the Primer-BLAST tool from NCBI or from a previous publication [25]. Primers were obtained from Eurofins Italia, Torino, Italy; sequences are displayed in Table 1. Data were analysed using the Delta-Delta-Ct method, and were converted to a relative ratio (2^−DDCt^) for statistical analysis [77] by normalizing to the endogenous reference gene *Gapdh*, following previous studies [25]. The specificity of amplification products was evaluated by building a dissociation curve in the 60–95 °C range.

### 4.4. Protein Levels

Dissected brain regions were homogenised in N-Per Neuronal Protein Extraction Reagent (Thermo Fisher Scientific, Waltham, MA, USA) added with Pierce Protease and Phosphatase Inhibitor Mini Tablets (Thermo Fisher Scientific, Waltham, MA, USA) in glass homogenisers, incubated 10 min on ice and centrifuged at 10,000 g for 10 min at 4 °C. Protein concentration was measured on supernatants with the BCA assay kit (Thermo Fisher Scientific, Waltham, MA, USA). Twenty µg proteins were separated on 4–15% precast polyacrylamide gels (Bio-Rad, Hercules, CA, USA) in Mini-Protean Electrophoresis Cells (Bio-Rad, Hercules, CA, USA), transferred on Immun-Blot PVDF membranes (Bio-Rad, Hercules, CA, USA) in Mini Trans-Blot Transfer Cells (Bio-Rad, Hercules, CA, USA), and blocked in 5% skimmed milk in 0.1% Tween20 Tris-buffered saline (Merck). Incubation with primary antibodies was carried out overnight at 4 °C (Table 2); membranes were washed, incubated with the secondary antibody (Table 2), and revealed and quantified by enhanced chemiluminescence (Clarity ECL Substrate, Bio-Rad, Hercules, CA, USA) in a ChemiDoc instrument (Bio-Rad, Hercules, CA, USA) using the Quantity One 1-D Analysis software (Bio-Rad, Hercules, CA, USA).

### 4.5. Statistical Analysis

Group size was 6–7 rats/group. The results were analysed using a 1-way ANOVA approach with treatment as the factor of interest, followed by Planned Comparisons of the predicted means. The data were log-transformed when needed in order to stabilise the variance and satisfy the parametric assumptions. An additional blocking factor Plate was also included in the model to account for any plate-to-plate variability as samples were analysed in different plates using a complete block design [78]; the same approach was adopted for Western blot gels. The data are shown as least square predicted means with 95% confidence intervals. A value of *p* < 0.05 was considered statistically significant. Multivariate analysis was carried out with Principal Components Analysis (PCA) with the objective of explaining most variability with a reduced number of components identified in unsupervised manner. All analyses were performed using the InVivoStat v4.4.0 software [78,79].

## Figures and Tables

**Table 1:** Primer sequences.

Gene	Forward Primer	Reverse Primer	Tm	Product Size
*Bmal1*	TGCCACTGACTACCAAGAAAGT	TGTCCCGACGCCTCTTTTCA	59.56 61.75	134
*Bdnf*	AAGTCTGCATTACATTCCTCGA	GTTTTCTGAAAGAGGGACAGTTTAT	57.53 57.49	138 [25]
*Creb*	CAGACAACCAGCAGAGTGGA	CTGGACTGTCTGCCCATTG	59.61 58.14	176
*Fgf-2*	GCGAACCGGTACCTGGCTAT	GCCCAGTTCGTTTCAGTGCC	62.02 62.13	160
*Fgfr2*	CCGGCCCTCCTTCAGTTTAG	TTCAACATGCAGCGCAACTC	60.11 60.04	132
*Gapdh*	AGACAGCCGCATCTTCTTGT	CTTGCCGTGGGTAGAGTCAT	59.68 59.46	207 [25]
*Per1*	GCGTTGCAAACGGGATGT	GCAGGCGAGATGGTGTAGTAGA	59.36 61.59	101
*Per2*	TGACGGGTCGAGCAAAGGAC	CCACGTCTTCCTGGAGCACA	62.43 62.10	163
*TrkB*	AAGTTCTACGGTGTCTGTGTG	TTCTCTCCTACCAAGCAGTTC	57.91 57.04	257 [25]

**Table 2:** Antibody list.

Antibody	Dilution	Cat. Number	RRID	Reactivity	Manufacturer
anti-phospho CREB Ser 133	1:1000	06-519	AB_310153	mouse, rat, hamster, human	Millipore, Burlington, MA, USA
anti-CREB	1:1000	9197	AB_331277	human, mouse, rat, monkey, D. melanogaster	Cell Signaling, Danvers, MA, USA
anti-Gapdh	1:1000	MA5-15738	AB_10977387	bacteria, canine, chicken, hamster, human, insect, mouse, rabbit, rat, yeast	Thermo Fisher Scientific, Waltham, MA, USA
Donkey Anti-rabbit IgG	1:3000	NA934	AB_772206	rabbit	GE Healthcare, Chicago, IL, USA

## Data Availability

Data is contained within the article or Supplementary Material.

## Supplementary Materials

The following supporting information can be downloaded at: https://www.mdpi.com/article/10.3390/ijms232214041/s1.
