# Supplementary material for: Chronic Trazodone and Citalopram Treatments Increase Trophic Factor and Circadian Rhythm Gene Expression in Rat Brain Regions Relevant for Antidepressant Efficacy
Source: Int J Mol Sci. 2022 Nov 14;23(22):14041. doi: 10.3390/ijms232214041 (PMC9698904; doi:10.3390/ijms232214041)
Supplement: Supplementary file 1 [file ijms-23-14041-s001.zip › Carboni et al Supplementary figure S1.pdf]

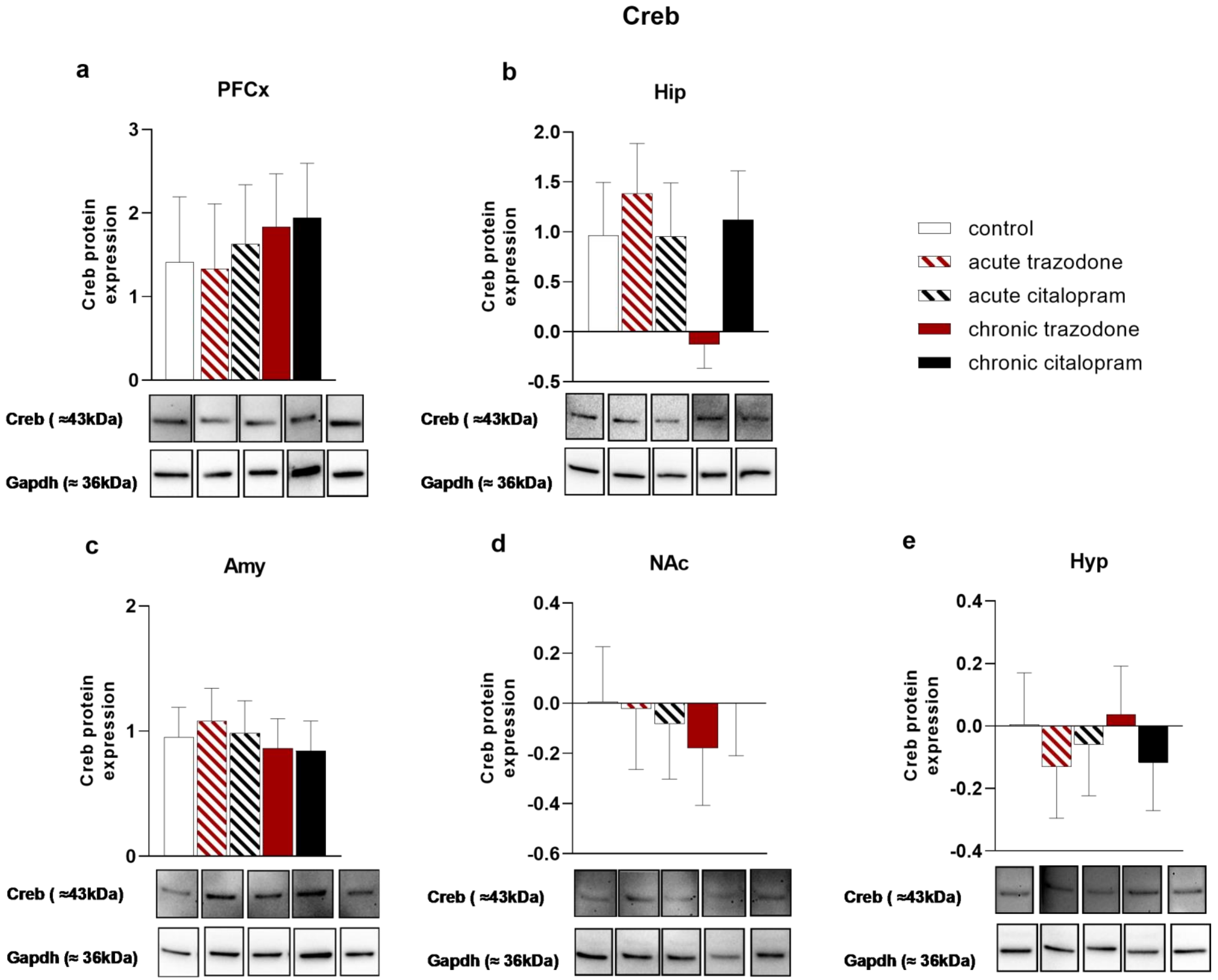

Supplementary Figure S1 Protein levels of Creb in the PFCx (a), Hip (b), Amy (c), NAc (d), Hyp (e) after acute or chronic treatment with trazodone, citalopram, or vehicle. The least square predicted means with 95% confidence intervals are plotted. Data were log- transformed when required to stabilize the variance and satisfy the parametric assumptions (d, e). N=6/group. Representative blots are shown in each panel; blot images are displayed in Supplementary Figure S2.
