# Supplementary material for: Chronic Trazodone and Citalopram Treatments Increase Trophic Factor and Circadian Rhythm Gene Expression in Rat Brain Regions Relevant for Antidepressant Efficacy
Source: Int J Mol Sci. 2022 Nov 14;23(22):14041. doi: 10.3390/ijms232214041 (PMC9698904; doi:10.3390/ijms232214041)

Supplementary Figure S2 Original western blot images

Amygdala (Amy)

ctrl; ac traz; ac cit; chr traz; chr cit; ctrl; ac traz; ac cit; chr traz

Ph-Creb

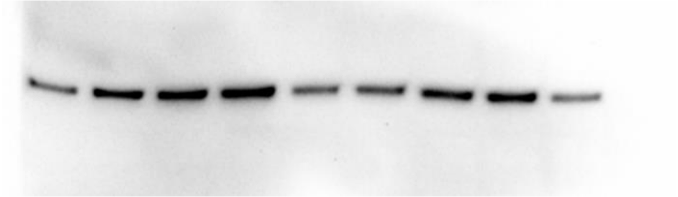

ctrl; ac traz; ac cit; chr traz; chr cit; ctrl; ac traz; ac cit; chr traz

Creb

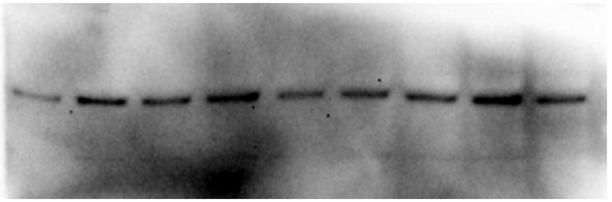

ctrl; ac traz; ac cit; chr traz; chr cit; ctrl; ac traz; ac cit; chr traz

Gapdh

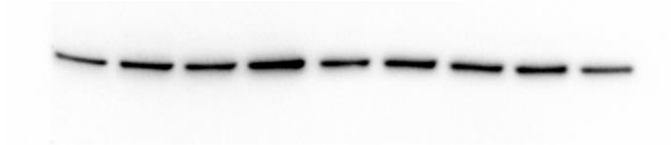

Amygdala (Amy)

chr traz; chr cit; ctrl; ac traz; ac cit; chr traz; chr cit; ctrl;

Ph-Creb

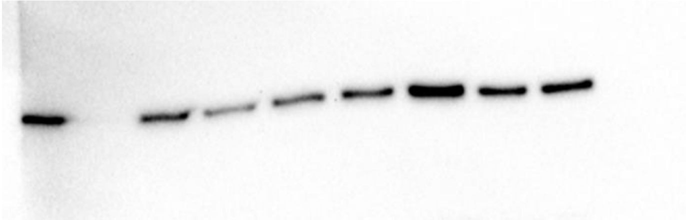

chr traz; chr cit; ctrl; ac traz; ac cit; chr traz; chr cit; ctrl;

Creb

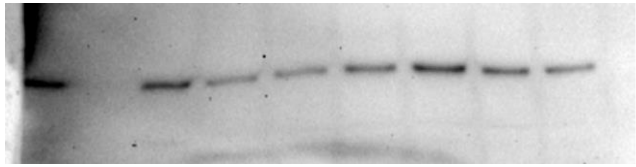

chr traz; chr cit; ctrl; ac traz; ac cit; chr traz; chr cit; ctrl;

Gapdh

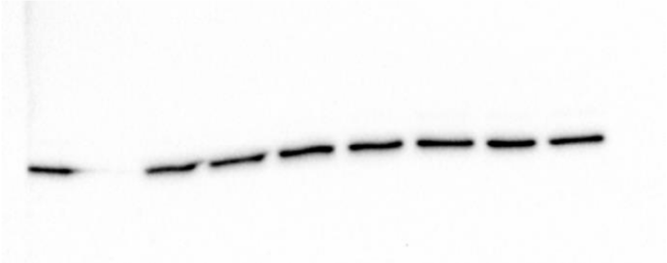

Amygdala (Amy)

ac traz; ac cit; chr traz; chr cit; ctrl; ac traz; ac cit; chr traz; chr cit

Ph-Creb

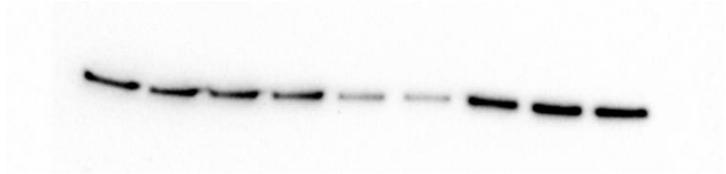

ac traz; ac cit; chr traz; chr cit; ctrl; ac traz; ac cit; chr traz; chr cit

Creb

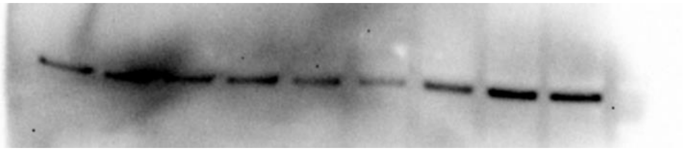

ac traz; ac cit; chr traz; chr cit; ctrl; ac traz; ac cit; chr traz; chr cit

Gapdh

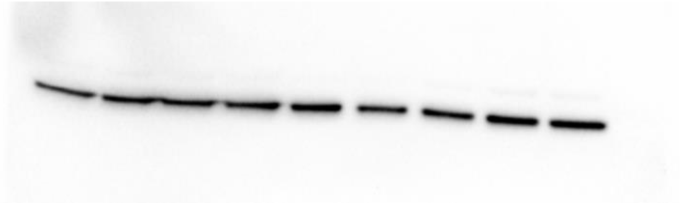

Amygdala (Amy)

ctrl; ac traz; ac cit; chr traz; chr cit; ctrl; chr traz; chr traz; chr cit

Ph-Creb

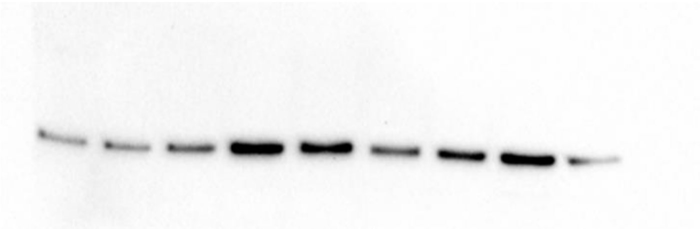

ctrl; ac traz; ac cit; chr traz; chr cit; ctrl; chr traz; chr traz; chr cit

Creb

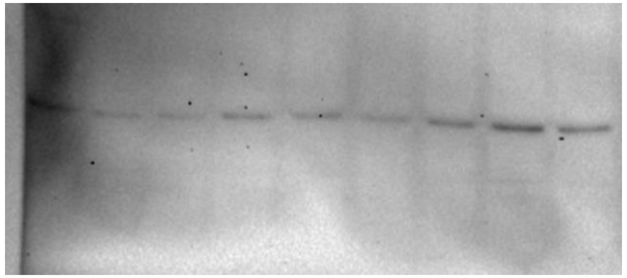

ctrl; ac traz; ac cit; chr traz; chr cit; ctrl; chr traz; chr traz; chr cit

Gapdh

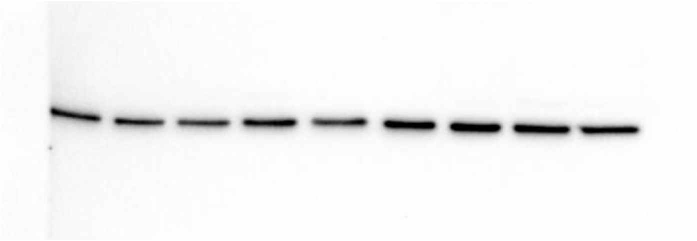

Hippocampus (Hip)

ctrl; ac traz; ac cit; chr traz; chr cit; ctrl; ac traz; ac cit; chr traz

Ph-Creb

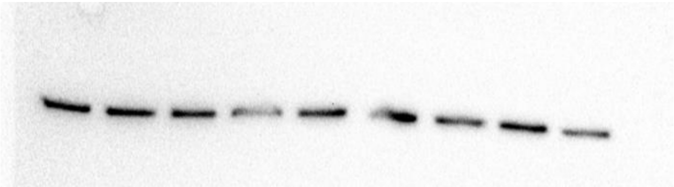

ctrl; ac traz; ac cit; chr traz; chr cit; ctrl; ac traz; ac cit; chr traz

Creb

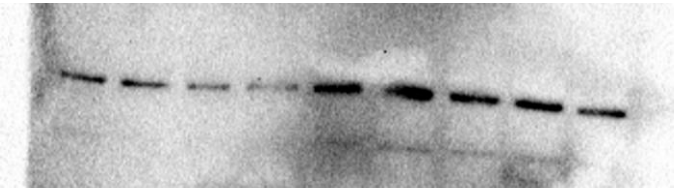

ctrl; ac traz; ac cit; chr traz; chr cit; ctrl; ac traz; ac cit; chr traz

Gapdh

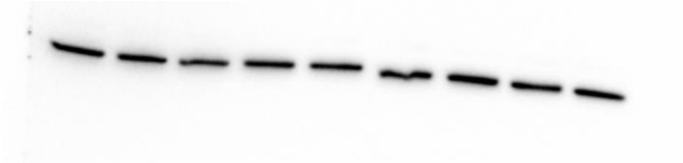

Hippocampus (Hip)

chr traz; chr cit; ctrl; ac traz; ac cit; chr traz; chr cit; ctrl; chr cit

Ph-Creb

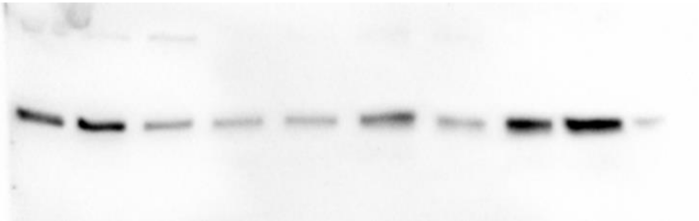

chr traz; chr cit; ctrl; ac traz; ac cit; chr traz; chr cit; ctrl; chr cit

Creb

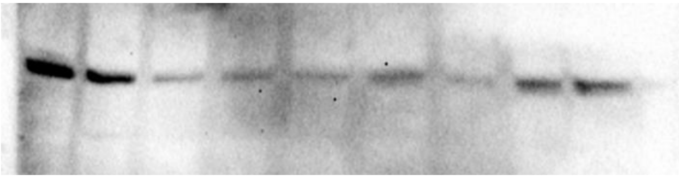

chr traz; chr cit; ctrl; ac traz; ac cit; chr traz; chr cit; ctrl; chr cit

Gapdh

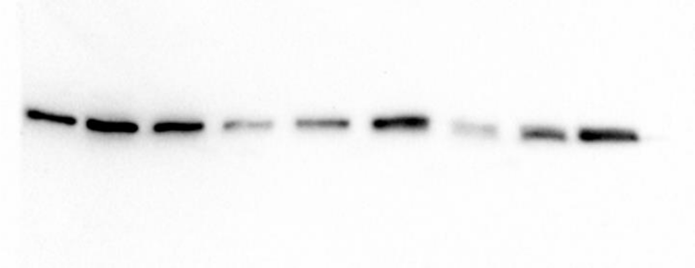

Hippocampus (Hip)

ac traz; ac cit; chr traz; chr cit; ctrl; ac traz; ac cit; chr traz; chr cit

Ph-Creb

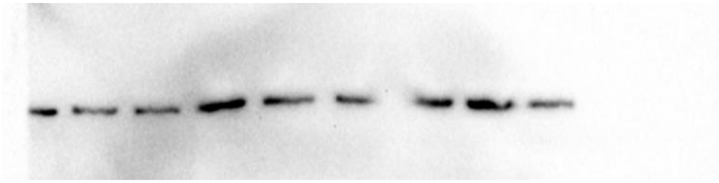

ac traz; ac cit; chr traz; chr cit; ctrl; ac traz; ac cit; chr traz; chr cit

Creb

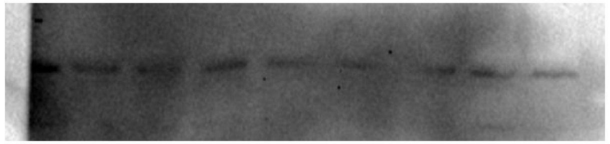

ac traz; ac cit; chr traz; chr cit; ctrl; ac traz; ac cit; chr traz; chr cit

Gapdh

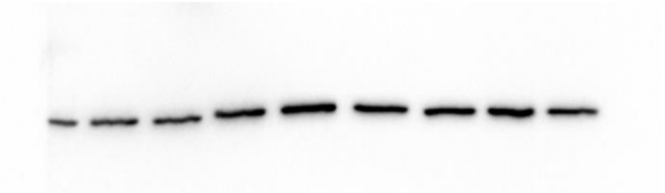

Hippocampus (Hip)

ctrl; ac traz; ac cit; chr traz; chr cit; ctrl; ac cit; chr traz; chr traz

Ph-Creb

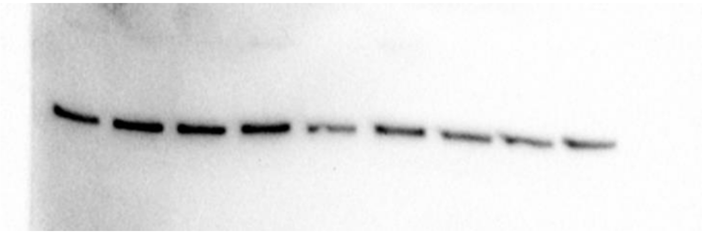

ctrl; ac traz; ac cit; chr traz; chr cit; ctrl; ac cit; chr traz; chr traz

Creb

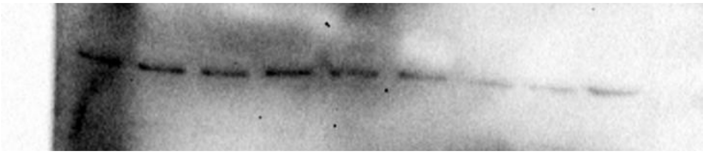

ctrl; ac traz; ac cit; chr traz; chr cit; ctrl; ac cit; chr traz; chr traz

Gapdh

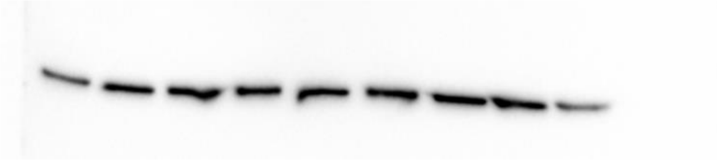

Nucleus Accumbens (NAc)

ctrl; ac traz; ac cit; chr traz; chr cit; ctrl; ac traz; ac cit; chr traz

Ph-Creb

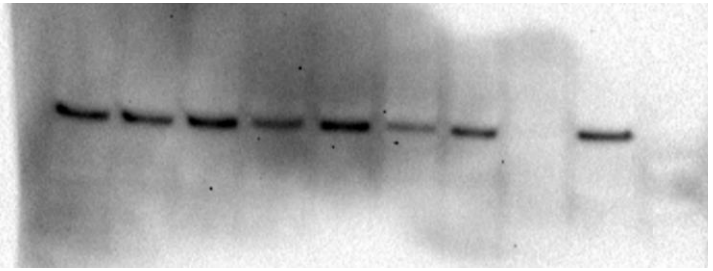

ctrl; ac traz; ac cit; chr traz; chr cit; ctrl; ac traz; ac cit; chr traz

Creb

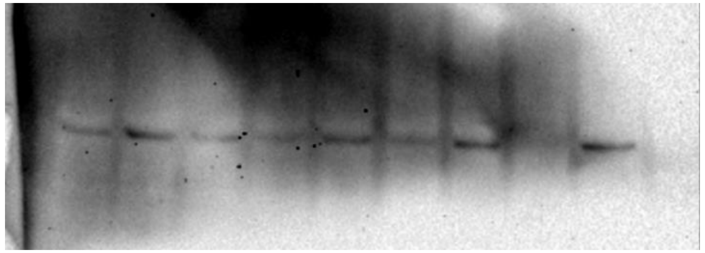

ctrl; ac traz; ac cit; chr traz; chr cit; ctrl; ac traz; ac cit; chr traz

Gapdh

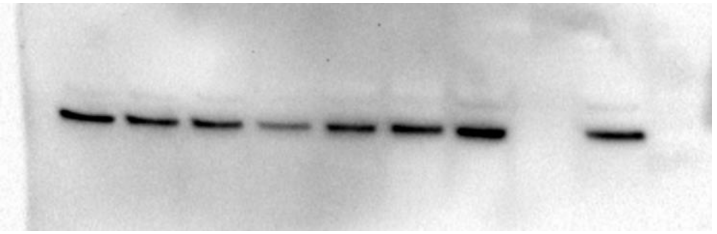

Nucleus Accumbens (NAc)

chr traz; chr cit; ctrl; ac traz; ac cit; chr traz; chr cit; ctrl; chr cit

Ph-Creb

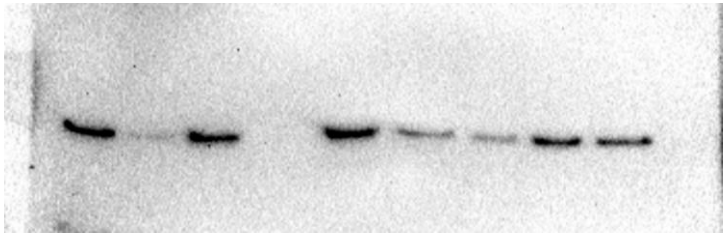

chr traz; chr cit; ctrl; ac traz; ac cit; chr traz; chr cit; ctrl; chr cit

Creb

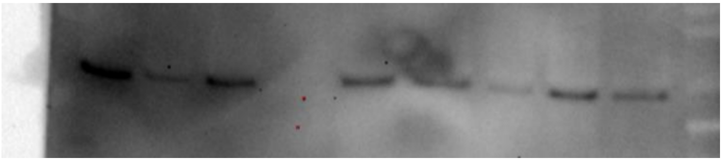

chr traz; chr cit; ctrl; ac traz; ac cit; chr traz; chr cit; ctrl; chr cit

Gapdh

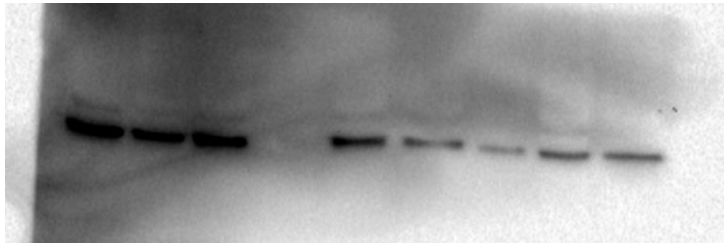

Nucleus Accumbens (NAc)

ac traz; ac cit; chr traz; chr cit; ctrl; ac traz; ac cit; chr traz; chr cit

Ph-Creb

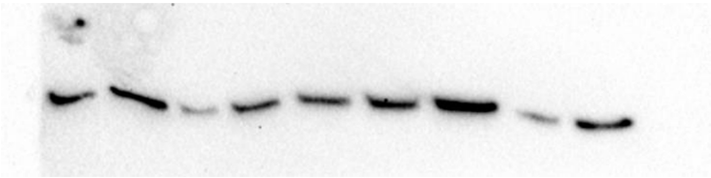

ac traz; ac cit; chr traz; chr cit; ctrl; ac traz; ac cit; chr traz; chr cit

Creb

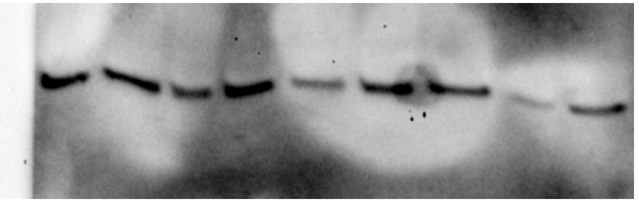

ac traz; ac cit; chr traz; chr cit; ctrl; ac traz; ac cit; chr traz; chr cit

Gapdh

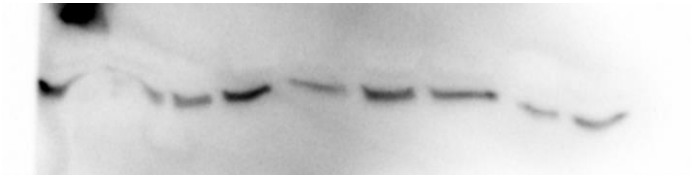

Nucleus Accumbens (NAc)

ctrl; ac traz; ac cit; chr traz; chr cit; ac traz; ac cit; ac traz; chr traz

Ph-Creb

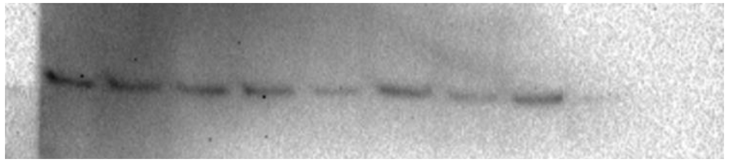

ctrl; ac traz; ac cit; chr traz; chr cit; ac traz; ac cit; ac traz; chr traz

Creb

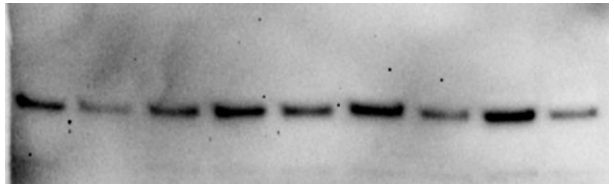

ctrl; ac traz; ac cit; chr traz; chr cit; ac traz; ac cit; ac traz; chr traz

Gapdh

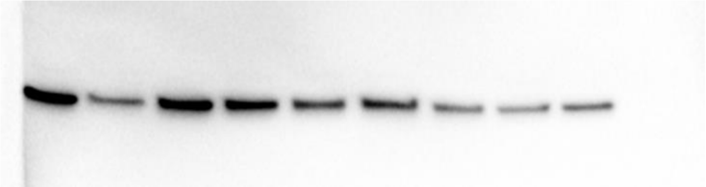

Hypothalamus (Hyp)

ctrl; ac traz; ac cit; chr traz; chr cit; ctrl; ac traz; ac cit; chr traz

Ph-Creb

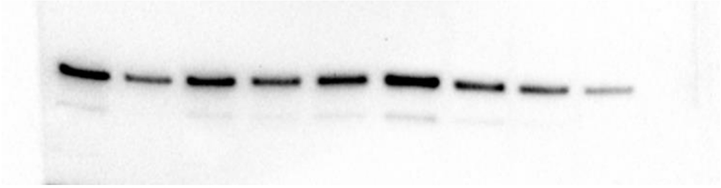

ctrl; ac traz; ac cit; chr traz; chr cit; ctrl; ac traz; ac cit; chr traz

Creb

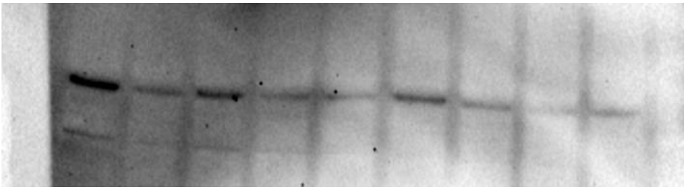

ctrl; ac traz; ac cit; chr traz; chr cit; ctrl; ac traz; ac cit; chr traz

Gapdh

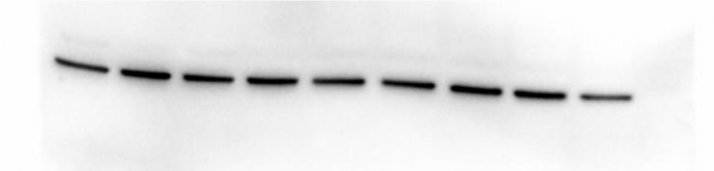

Hypothalamus (Hyp)

chr traz; chr cit; ctrl; ac traz; ac cit; chr traz; chr cit; ctrl; chr cit

Ph-Creb

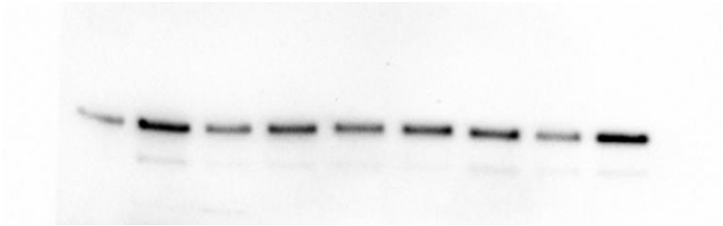

chr traz; chr cit; ctrl; ac traz; ac cit; chr traz; chr cit; ctrl; chr cit

Creb

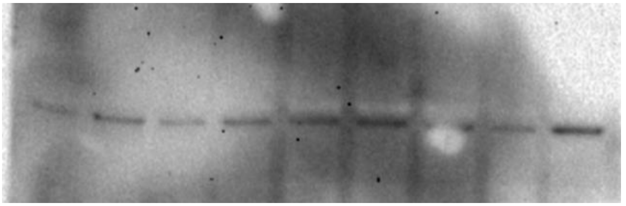

chr traz; chr cit; ctrl; ac traz; ac cit; chr traz; chr cit; ctrl; chr cit

Gapdh

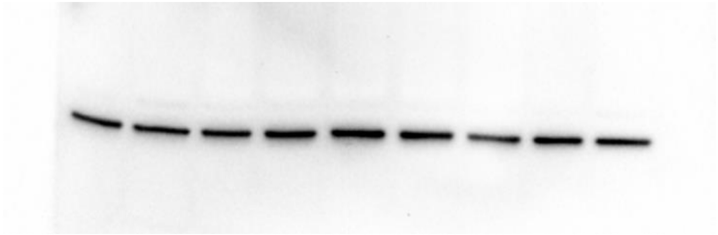

Hypothalamus (Hyp)

ac traz; ac cit; chr traz; chr cit; ctrl; ac traz; ac cit; chr traz; chr cit

Ph-Creb

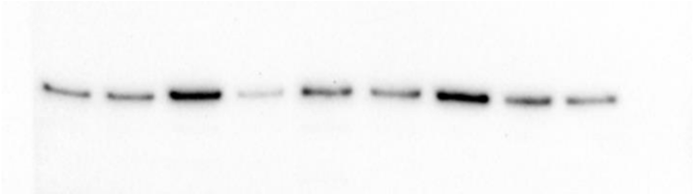

ac traz; ac cit; chr traz; chr cit; ctrl; ac traz; ac cit; chr traz; chr cit

Creb

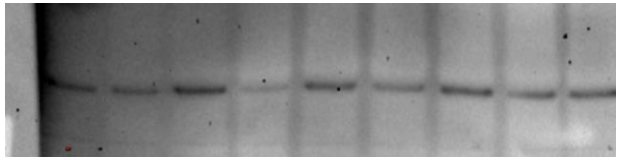

ac traz; ac cit; chr traz; chr cit; ctrl; ac traz; ac cit; chr traz; chr cit

Gapdh

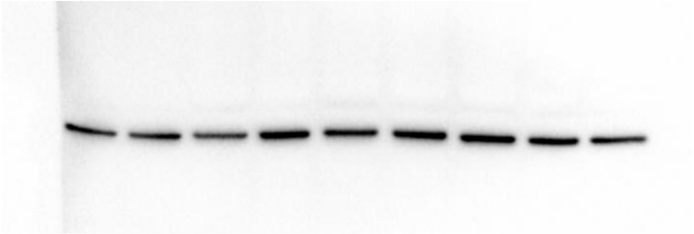

Hypothalamus (Hyp)

ctrl; ac traz; ac cit; chr traz; chr cit; ctrl; ac cit; chr traz; chr traz

Ph-Creb

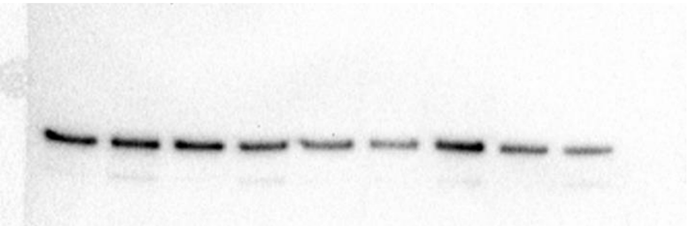

ctrl; ac traz; ac cit; chr traz; chr cit; ctrl; ac cit; chr traz; chr traz

Creb

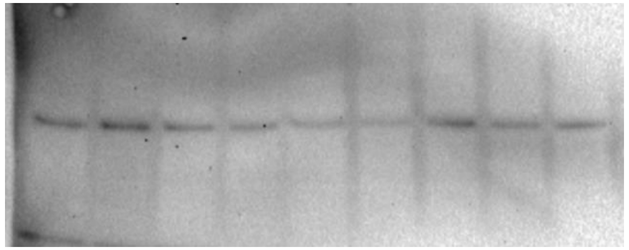

ctrl; ac traz; ac cit; chr traz; chr cit; ctrl; ac cit; chr traz; chr traz

Gapdh

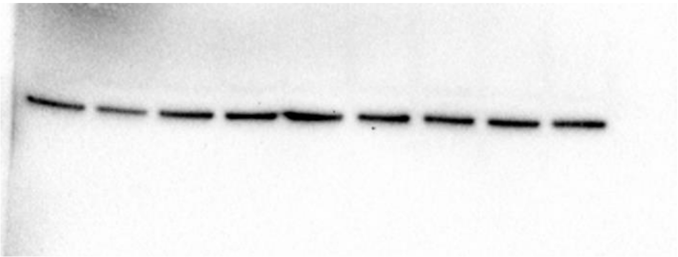

Pre-frontal cortex (PFCx)

ctrl; ac traz; ac cit; chr traz; chr cit; ctrl; ac traz; ac cit; chr traz

Ph-Creb

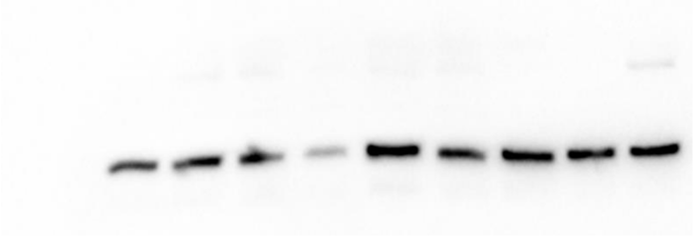

ctrl; ac traz; ac cit; chr traz; chr cit; ctrl; ac traz; ac cit; chr traz

Creb

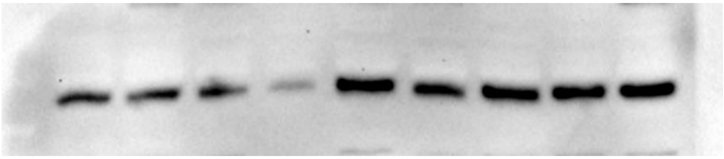

ctrl; ac traz; ac cit; chr traz; chr cit; ctrl; ac traz; ac cit; chr traz

Gapdh

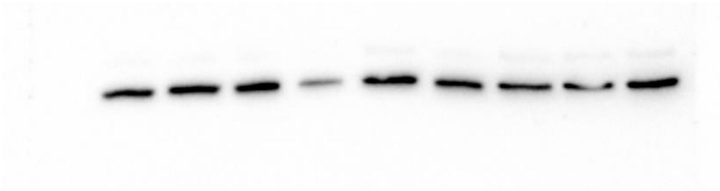

Pre-frontal cortex (PFCx)

chr traz; chr cit; ctrl; ac traz; ac cit; chr traz; chr cit; ctrl; chr cit

Ph-Creb

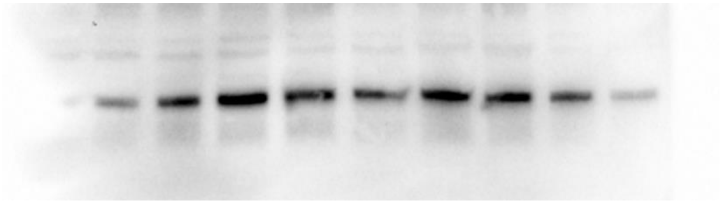

chr traz; chr cit; ctrl; ac traz; ac cit; chr traz; chr cit; ctrl; chr cit

Creb

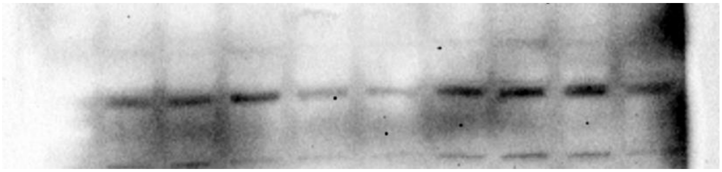

chr traz; chr cit; ctrl; ac traz; ac cit; chr traz; chr cit; ctrl; chr cit

Gapdh

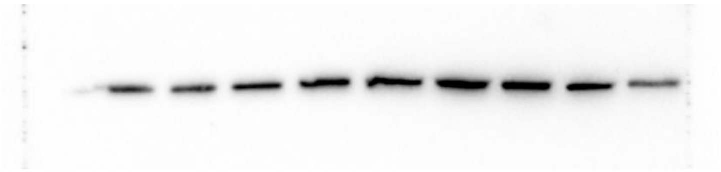

Pre-frontal cortex (PFCx)

ac traz; ac cit; chr traz; chr cit; ctrl; ac traz; ac cit; chr traz; chr cit

Ph-Creb

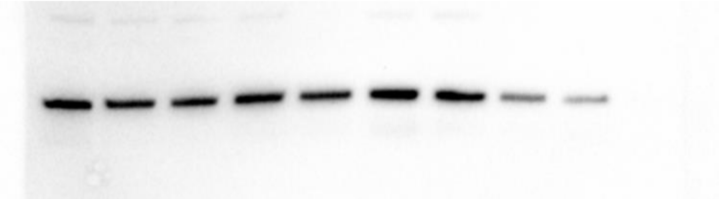

ac traz; ac cit; chr traz; chr cit; ctrl; ac traz; ac cit; chr traz; chr cit

Creb

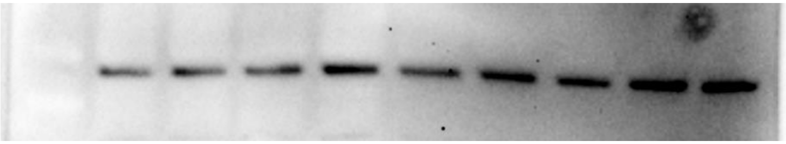

ac traz; ac cit; chr traz; chr cit; ctrl; ac traz; ac cit; chr traz; chr cit

Gapdh

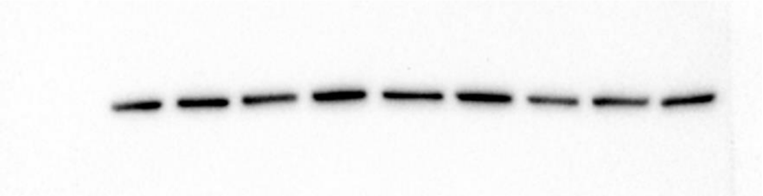

Pre-frontal cortex (PFCx)

ctrl; ac traz; ac cit; chr traz; chr cit; chr traz; ac traz; ac cit; chr traz

Ph-Creb

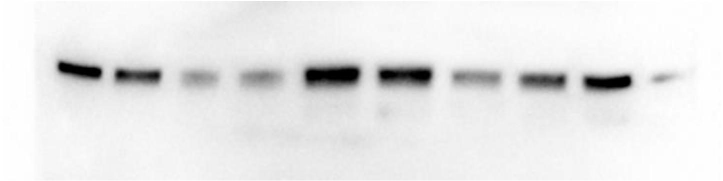

ctrl; ac traz; ac cit; chr traz; chr cit; chr traz; ac traz; ac cit; chr traz

Creb

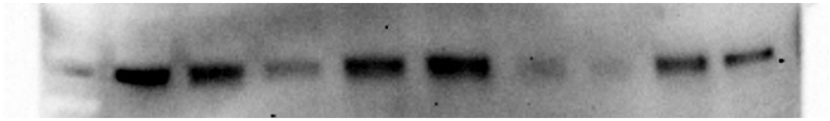

ctrl; ac traz; ac cit; chr traz; chr cit; chr traz; ac traz; ac cit; chr traz

Gapdh

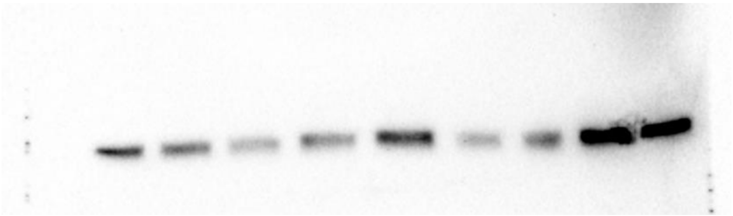

Supplement: Supplementary file 1 [file ijms-23-14041-s001.zip › Carboni et al Supplementary Figure S2.pdf]
