# Supplementary material for: Chronic Trazodone and Citalopram Treatments Increase Trophic Factor and Circadian Rhythm Gene Expression in Rat Brain Regions Relevant for Antidepressant Efficacy
Source: Int J Mol Sci. 2022 Nov 14;23(22):14041. doi: 10.3390/ijms232214041 (PMC9698904; doi:10.3390/ijms232214041)

Supplementary Figure S3 Experimental design

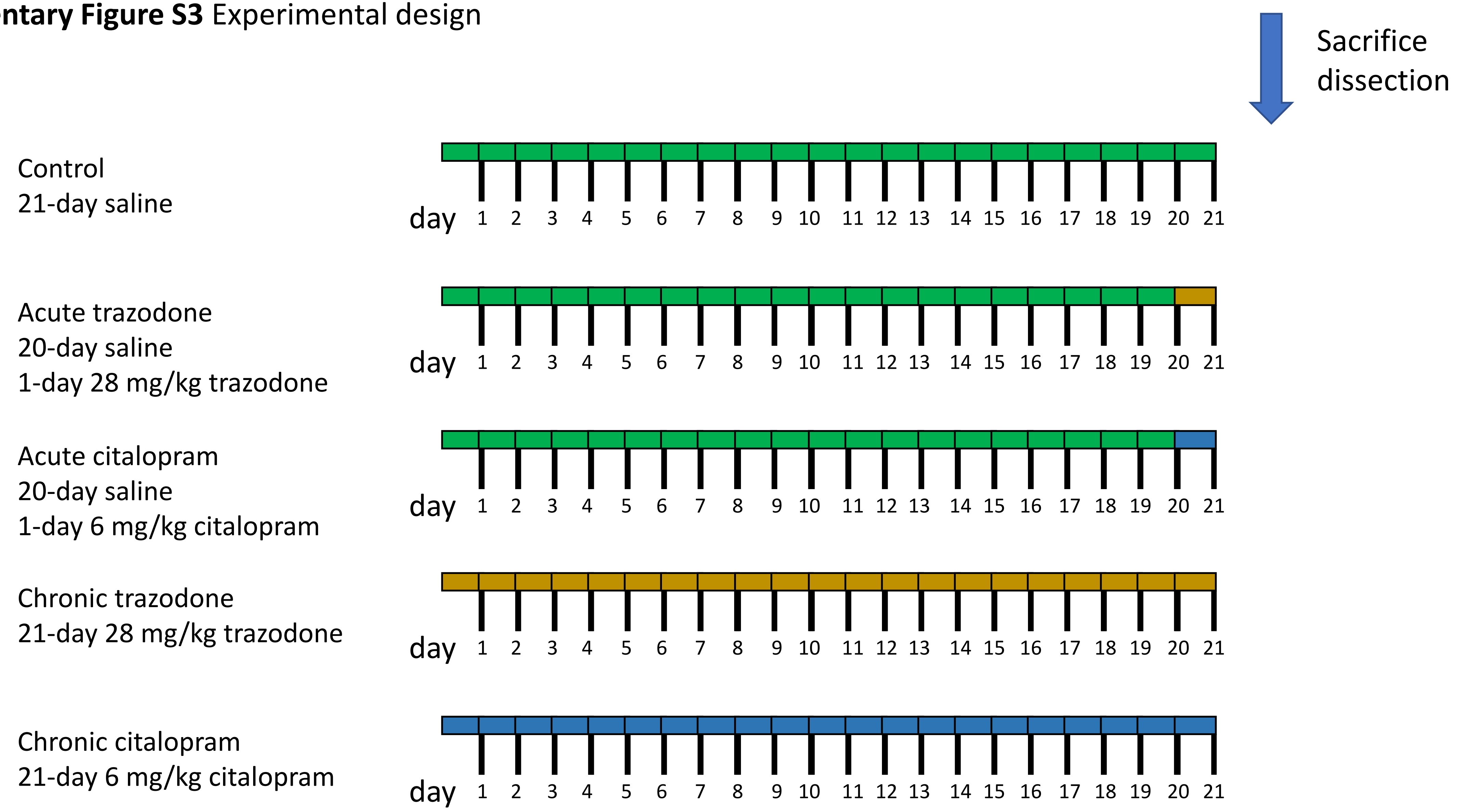

Supplement: Supplementary file 1 [file ijms-23-14041-s001.zip › Carboni et al Supplementary figure S3.pdf]
